# Exploring the potential of cranial non-metric traits as a tool for personal identification: the never-ending dilemma

**DOI:** 10.1007/s00414-021-02654-4

**Published:** 2021-07-17

**Authors:** Andrea Palamenghi, Alessia Borlando, Danilo De Angelis, Chiarella Sforza, Cristina Cattaneo, Daniele Gibelli

**Affiliations:** 1 LABANOF, Laboratorio di Antropologia e Odontologia Forense, Sezione di Medicina Legale, Dipartimento di Scienze Biomediche per la Salute, Università degli Studi di Milano, Via L. Mangiagalli 37, 20133 Milan, Italy; 2 LAFAS, Laboratorio di Anatomia Funzionale dell’Apparato Stomatognatico, Dipartimento di Scienze Biomediche per la Salute, Università degli Studi di Milano, Via L. Mangiagalli 31, 20133 Milan, Italy

**Keywords:** Anatomical variants, Cranium, Frequencies, Personal identification, Unknown skeletal remains

## Abstract

Forensic anthropologists tasked with identification of skeletal remains often have to set up new strategies to overcome the limitations of conventional individualizing markers. A sound acquaintance with non-metric traits is essential for a reliable distinction between normal variations and pathological or traumatic conditions, yet the role of cranial variants in the identification process is still somehow ill-defined. One hundred crania (50 males and 50 females) of known sex and age were selected from the Collezione Antropologica LABANOF (a documented contemporary skeletal collection) and non-metric traits were scored as present or absent and by side. The frequencies of 13 traits were used to calculate the compound probabilities to find an individual with an exact combination of cranial features in the worldwide population. The probabilities of the majority of the individuals (53%) are within the 1 out of 10 million–1 out of 1 million interval. However, a fair number of subjects (25%) of the sample have the probabilities falling into the 1 out of 1 billion–1 out of 100 million interval, while the probabilities of a small portion of the sample (10%) are less than 1 out of 1 billion. This pilot study illustrates that some combinations of cranial variants are quite rare and may represent potential evidence to discern presumptive identifications, when an appropriate set of traits is selected and antemortem data are available for comparison. However, further research on larger and various samples is needed to confirm or discard the use of combinations of cranial non-metric traits as individualizing markers.

## Introduction

Personal identification of human remains is a fundamental human right that must be upheld for legal, administrative, social, and psychological reasons [1, 2]. This process relies first upon unique biological evidence that helps discern an individual from another, e.g., physical and genetic data acquired from a postmortem (PM) examination [3]. Forensic anthropologists tasked with identification of skeletal remains investigate extrinsic (e.g., evidence of surgical procedures and orthopedic devices) or intrinsic (e.g., peculiarities, anomalies, and anatomical variants) features of the skeleton [4–7] that can subsequently be compared with antemortem (AM) data to establish a conclusive identification. Examination of the human cranium is an essential step of the biological profile of unknown skeletal remains, which may eventually provide significant information for personal identification. In particular, morphological analyses are required when metric evaluations cannot be performed due to the poor state of preservation of the remains (e.g., fragmentation, incompleteness) [8]. In addition to the morphological features extensively used for sex and ancestry estimation [9, 10], the human cranium holds an enormous array of anatomical variants that are known as non-metric (or discontinuous/discrete/epigenetic) traits. These present as a wide range of differences in the morphology and number of foramina, tubercles, ossicles, grooves, and sutures [11] and they usually represent deviations from the normal skeletal development [12]. Berry and Berry [13] first investigated the incidence of non-metric traits of the human cranium in eight different skeletal populations from archeological settings. Thereon, non-metric variants have been used to calculate the biological distance between populations and relatedness between individuals [13–16]. In forensic anthropology, a sound acquaintance with non-metric traits is essential to differentiate normal from pathological anatomy, as some traits can mimic pathological conditions or traumatic injuries of the skeleton [11, 17]. In clinical medicine, variability of these traits among individuals influences surgical procedures, i.e., the correct localization of foramina for the passage of neurovascular structures might prevent damage when anesthesia is performed [18]. Traditionally, unique cranial morphological features useful to personal identification include paranasal sinuses (e.g., frontal sinuses), that have proved to be substantially diversified among individuals and therefore a reliable mean for identification [19]. Apart from dental features, other cranial peculiarities that have been suggested for this purpose include endocranial arterial [20] and suture patterns [21, 22]. Non-metric traits of the postcranial skeleton have been used as well in personal identification via comparison of PM findings and AM radiographic images [5, 23, 24]. Although anthropologists record anatomical variants of the skeleton, such as accessory foramina and tubercles, peculiar exostosis (tori), and vascular grooves, in their standard analysis of skeletal remains and they potentially represent individualizing markers [23], the possible use of cranial non-metric traits to the personal identification procedure has not been explored yet. This study investigates the potential use of anatomical variants of the cranium for identification purposes, by calculating the probabilities to find an individual with a combination of cranial features, on the base of their prevalence in general population. The aim is to verify the potential rule of these variants in preliminary steps of personal identification, through the comparison between a set of traits recorded postmortem and similar data assessed antemortem through CT scan or conventional X-ray. A sample of 100 crania from an Italian contemporary documented skeletal collection was examined to detect non-metric traits. The frequencies of different sets of anatomical variants were assessed using a group of 13 variants, potentially assessable on CT scan: the same procedure was repeated for a subset of seven variants which may be recorded also on conventional X-ray. In addition, frequencies of the traits in this population are presented by side and sex. These variants could represent an additional tool that may help forensic anthropologists elaborate a comprehensive biological profile, providing supplementary information for a positive identification of unknown skeletal remains, when appropriate AM material is available.

**Table 1:** Anatomical variants considered to calculate the probabilities to find a combination of traits in the general population

Non-metric traits	Position	Description	Evaluated features
1. Supraorbital foramen	Frontal bone	Complete foramen on the supraorbital ridge	P/A
2. Accessory supraorbital foramen	Frontal bone	Accessory foramina to the one on the margin	P/A of multiple foramina
3. Infraorbital foramen	Maxillary bone	Foramen on the anterior surface of the bone	P/A of multiple foramina
4. Lesser palatine foramina	Palatine bone	One or several foramina posterior to the greater palatine foramen	Number of foramina
5. Palatine torus	Palatine bone	Bony protuberance along the median palatine suture	P/A
6. Maxillary torus	Maxillary bone	Bony protuberance on the palatal surface of the molar alveoli	P/A
7. Mandibular torus	Mandible	Bony protuberance on the lingual surface of the mandible	P/A
8. Paracondylar foramina	Occipital bone	Foramina located laterally to the occipital condyles	P/A
9. Hypoglossal canal	Occipital bone	Canal perforating the base of the occipital condyles	Single or double
10. Nasal foramina	Nasal	One or more foramina perforating the nasal bones	P/A
11. Parietal foramina	Parietal bone	One or several foramina perforating the bone in the obelion area	P/A
12. Paracondylar process	Occipital bone	Bony protrusion medial to the mastoid process, lateral to the occipital condyles and posterior to the jugular fossa	P/A
13. Precondylar process	Occipital bone	Thickenings on the anterior margin of the occipital foramen	P/A

*P/A* presence/absence of the trait. The traits are described according to Hauser and De Stefano [25]

## Material and methods

The skeletal remains under study are part of the Collezione Antropologica LABANOF (CAL) Milano Cemetery Skeletal Collection, which is hosted at the Laboratorio di Antropologia e Odontologia Forense (LABANOF) of the University of Milan (Italy). This collection is formed by the skeletons of over 2000 individuals that died between 1910 and 2000 and were collected from several cemeteries of Milan in accordance with the Italian Legislation. The article 43 of the Presidential Decree of the Italian Republic (DPR) no. 285 (September 10, 1990) allows the universities to collect unclaimed skeletal remains for educational and research purposes [26].

The study sample includes 100 crania equally distributed between males (N = 50) and females (*N* = 50). The individuals are aged between 24 and 94 years, with a mean age of 68.4 years and a standard deviation (SD) of 19.1 years. Males are aged between 24 and 90 years (mean age = 62.9; SD = 20.3). Females are aged between 24 and 94 years (mean age = 73.9; SD = 16). A Student t-test was run to assess age differences between males and females. Since these are dichotomous traits (thus repeatability would yield 100% of agreement), macroscopical observation of each cranium was concurrently performed by two operators with more than 10 years of experience in anthropology. The traits were scored by side as present or absent (Table 1), based on the consensus between the observers. The possible relationship between age and presence of traits was investigated. The sample was thus divided in three age groups (24–59 years, 60–80 years, > 80 years, Table 2) and a Chi-squared test (*p* < 0.01) was run for each trait. For the multiple lesser palatine foramina, representing an ordinal variable, this relationship was assessed through calculation of the Pearson’s correlation coefficient (*p* < 0.01). Moreover, the trend of all traits to be more represented monolaterally or bilaterally was explored through Chi-squared test (*p* < 0.01), using a conjectural sample with an equal distribution between monolateral and bilateral expression.

**Table 2:** Number of individuals per age group

Group	Age range	Males (N = 50)	Females (N = 50)
0	24–59	19	9
1	60–80	18	18
2	> 80	13	23

**Fig. 1:**
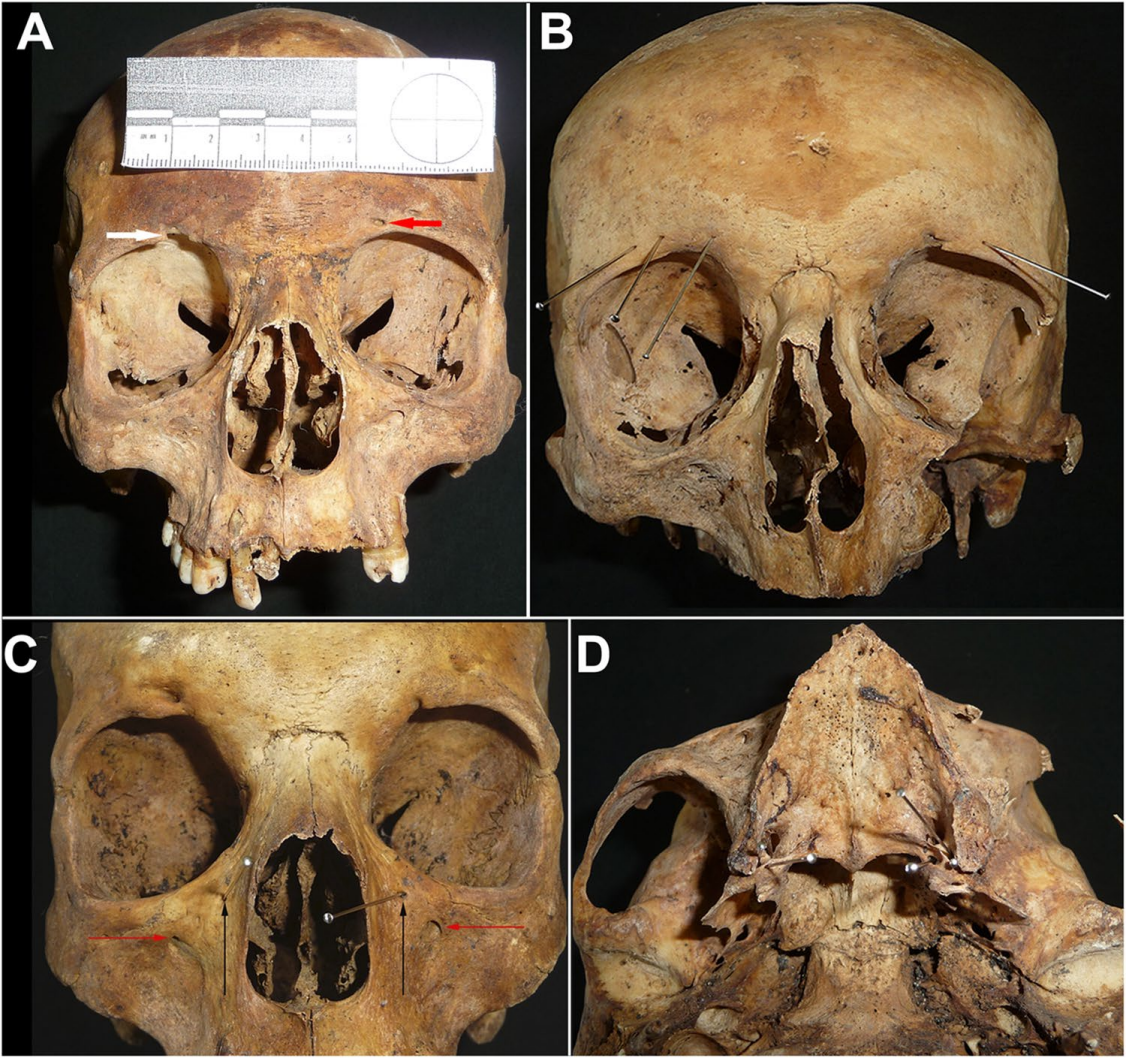
Non-metric traits considered for the calculation of the probabilities. **A** Shape of the supraorbital structure. The red arrow marks the supraorbital foramen, while the white arrow indicates the supraorbital notch. **B** Accessory supraorbital foramina. Note that there are three accessory foramina on the right supraorbital ridge and one on the left. **C** Multiple infraorbital foramina. The red arrow indicates the infraorbital foramen, and the black arrows mark the accessory infraorbital structures. **D** Lesser palatine foramina. As indicated by the pins, this individual had three lesser palatine foramina on the right-hand side and two on the left-hand side

**Fig. 2:**
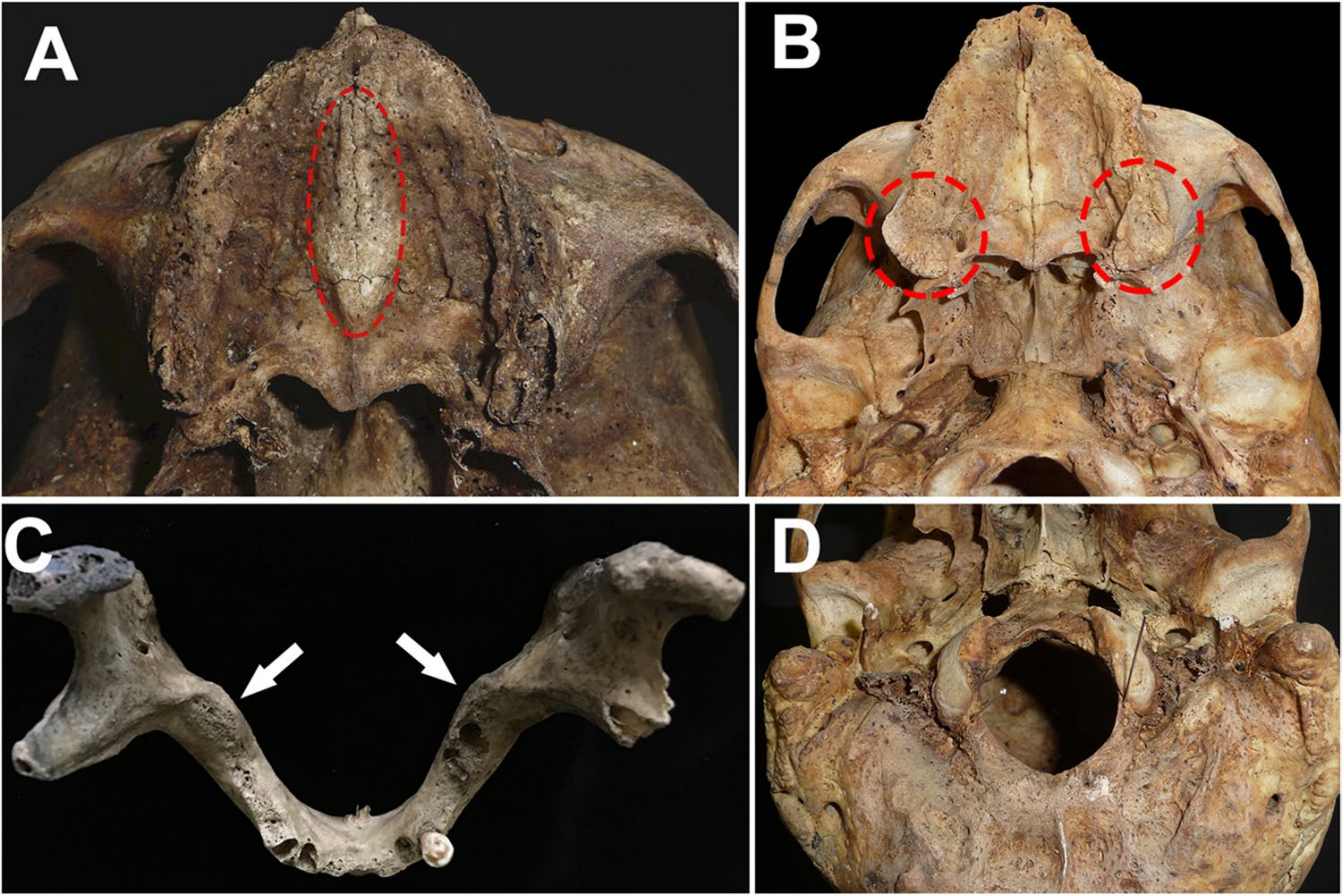
Non-metric traits considered for the calculation of the probabilities. **A** Palatine torus within the dotted red ellipse. **B** Maxillary tori within the dotted red circles. **C** Mandibular tori. The white arrows mark the position of the tori at the level of the lower third molar. **D** Paracondylar foramina. The pins locate the position of the foramina medial to the occipital condyles

**Fig. 3:**
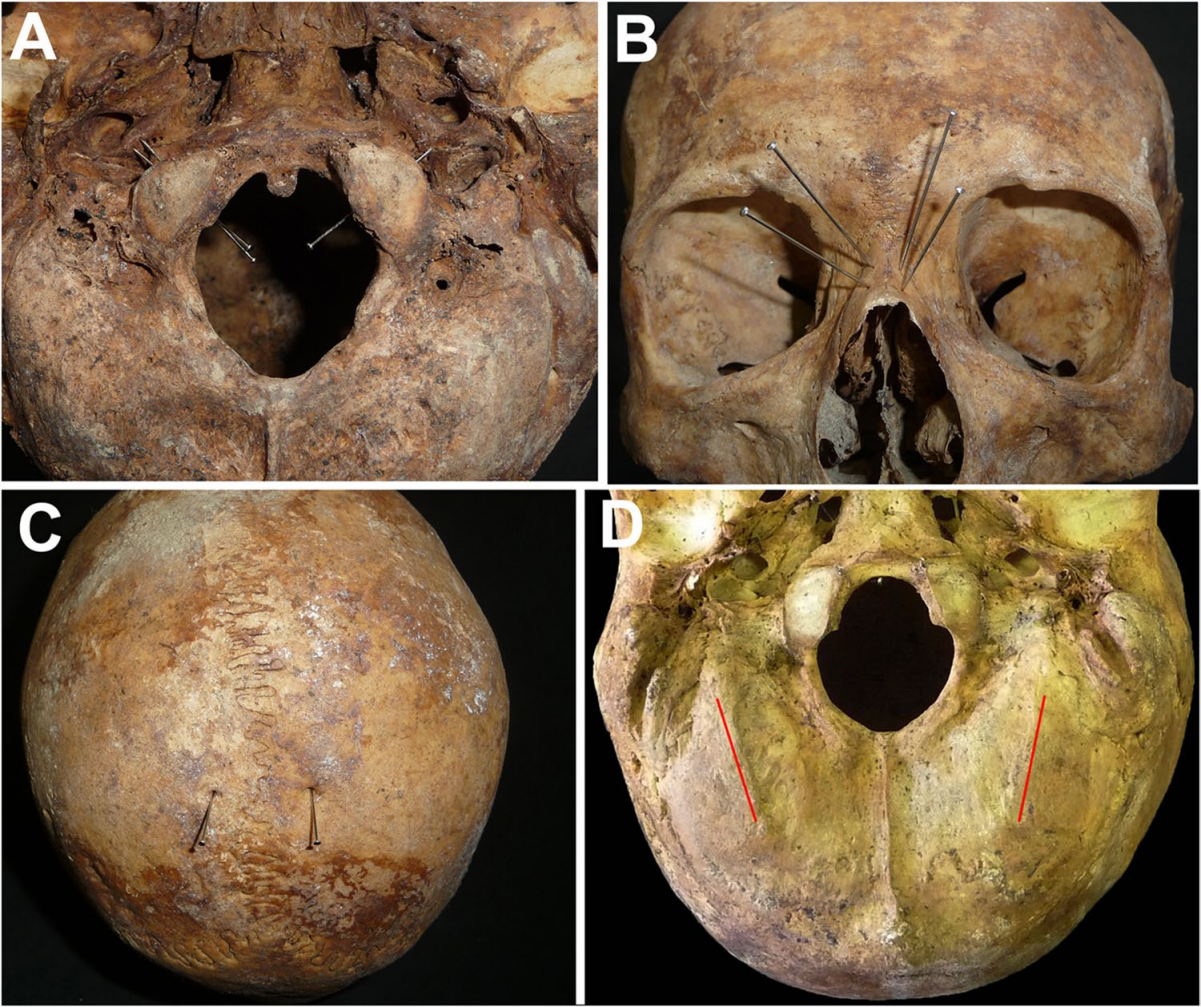
Non-metric traits considered for the calculation of the probabilities. **A** Hypoglossal canal. This cranium exhibits a double hypoglossal canal on the right-hand side and a single canal on the left-hand side. **B** Nasal foramina perforating the nasal bones, as indicated by the pins. **C** Parietal foramina, one on each side. **D** Paracondylar processes marked by the red lines

**Fig. 4:**
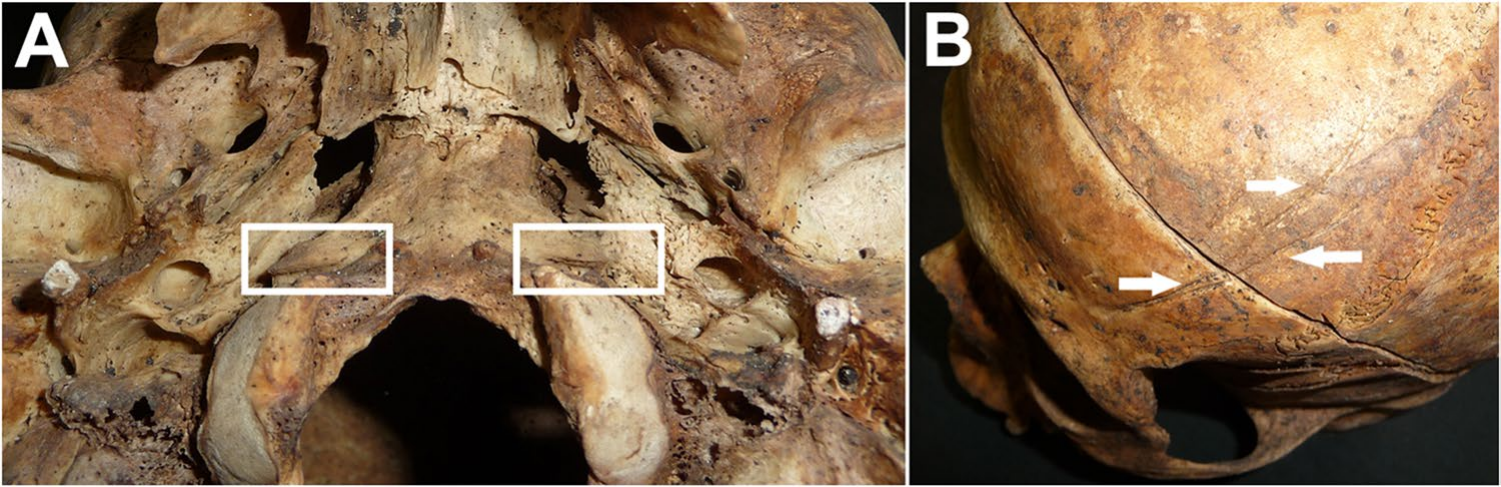
Non-metric traits considered for the calculation of the probabilities. **A** Precondylar processes inside the white rectangles. **B** Frontal grooves marked by the white arrows. Frontal grooves were initially considered for the calculation of probabilities, but they were discounted as the Chi-squared test showed a potential relation with age

Thirteen variants (12 bilateral and 1 median) were selected for the probability study (Table 1, Figs. 1, 2, 3, and 4) and the frequency of each trait in males and females was calculated and used as follows. For each subject, the 25 frequencies (12 right and 12 left, 1 median) served to calculate the compound probability: to wit, the product of the frequencies returned the probability that an individual presents an exact set of the traits taken into consideration. Each individual was then ranked based on the four different intervals of probability (< 1 out of 1 billion; 1 out of 1 billion–1 out of 100 million; 1 out of 10 million–1 out of 1 million; > 1 out of 1 million). In addition, the compound frequency of a selected subset of cranial traits potentially visible on a conventional X-ray (including supraorbital foramen, multiple infraorbital foramina, palatine, maxillary and mandibular tori, parietal foramina, and frontal grooves) was calculated for each individual.

## Results

No significant differences were found between males and females according to age (*p* > 0.05).

In males and females, no relationship between age and traits was found, except for the frontal grooves, but only in males and on the left side (*p* < 0.01). For this reason, the frequencies of frontal grooves were excluded from the calculation of the compound frequency. The analysis of monolateral and bilateral expression revealed that there is no tendency to bilaterality for most traits; only the mandibular torus and the paracondylar process were more frequently expressed bilaterally (*p* < 0.01). Considering the mandibular torus, most males presented a bilateral expression of the torus and only two male individuals presented the trait on one side (left). All females exhibited a bilateral expression. As for the paracondylar process, bilateral presence was observed in most males and females, with only three males and two females presenting monolateral expression. The frequencies of the variants considered for the calculation of probabilities are shown in Table 3. Overall, paracondylar foramina and the presence of four lesser palatine foramina were the least frequent traits both in males and females. High frequencies of palatine and mandibular tori were observed both in males and females, as well for the single hypoglossal canal. Table 4 summarizes the percentage of male and female individuals with different probabilities to have an exact mixture of cranial traits. On average, most individuals (53%) presented probabilities within the 1 out of 10 million–1 out of 1 million interval. Twenty-five percent of the sample presented probabilities at least of 1 out of 100 million. Interestingly, the probabilities of a small portion of the sample (10%) are lower than 1/1 billion, indicating that some combinations of traits are quite rare and may be potentially used for identification purposes. Table 5 reports four cases (two males and two females) that showed the lowest and highest compound frequencies, thus the lowest and highest probabilities to present a combination of traits.

**Table 3:** Frequencies of non-metric traits in the sample of 100 crania

Non-metric traits	Males (N = 50)		Females (N = 50)	
	Right	Left	Right	Left
Supraorbital foramen	0.22	0.18	0.22	0.24
Multiple supraorbital foramina	0.44	0.38	0.20	0.22
Multiple infraorbital foramina	0.18	0.20	0.12	0.14
Number of lesser palatine foramina				
1	0.20	0.34	0.35	0.34
2	0.35	0.45	0.46	0.45
3	0.35	0.19	0.15	0.19
4	0.09	0.02	0.02	0.02
Palatine torus	0.68		0.82	
Maxillary torus	0.40	0.44	0.26	0.28
Mandibular torus	0.72	0.76	0.80	0.80
Paracondylar foramina	0.08	0.10	0.08	0.06
Hypoglossal canal				
Single	0.82	0.74	0.84	0.62
Double	0.18	0.26	0.16	0.34
Nasal foramina	0.64	0.54	0.62	0.62
Parietal foramina	0.60	0.44	0.60	0.36
Paracondylar process	0.74	0.72	0.74	0.70
Precondylar process	0.36	0.42	0.24	0.24

**Table 4:** Percentage of individuals within classes of probability of finding an individual with a combination of the 13 anatomical variants

Class of probability	% males	% females	% total
< 1 out of 1 billion	10	10	10
1 out of 1 billion–1 out of 100 million	34	16	25
1 out of 10 million–1 out of 1 million	50	56	53
> 1 out of 1 million	6	18	12

**Table 5:** Individuals that showed the lowest and highest compound frequencies and whose probabilities to present such combination fall into the class < 1 out of 1 billion and > 1 out of 1 million, respectively

Non-metric traits	Males				Females			
	Individual 486		Individual 625		Individual 691		Individual 444	
	Right	Left	Right	Left	Right	Left	Right	Left
Supraorbital foramen	P	P	A	P	A	A	A	A
Multiple supraorbital foramina	A	P	P	P	A	A	A	A
Multiple infraorbital foramina	A	A	A	P	A	A	A	A
Number of lesser palatine foramina								
1	A	A	A	A	A	A	A	A
2	P	P	P	P	A	P	P	P
3	A	A	A	A	P	A	A	A
4	A	A	A	A	A	A	A	A
Palatine torus	P		P		P		P	
Maxillary torus	P	P	A	A	A	A	A	A
Mandibular torus	P	P	A	A	P	P	P	P
Paracondylar foramina	P	P	P	A	A	A	A	A
Hypoglossal canal								
Single	A	A	A	A	P	P	P	P
Double	P	P	P	P	A	A	A	A
Nasal foramina	A	A	P	P	P	P	P	P
Parietal foramina	A	A	P	A	P	P	A	A
Paracondylar process	P	P	A	P	P	P	P	P
Precondylar process	P	P	P	P	A	A	A	A
Compound frequency	0.00000000000637939		0.0000260071		0.00000000000010181		0.00021964	

*P* indicates presence of the trait, whereas *A* indicates the absence of the trait

When a subset of traits visible on conventional X-rays is considered, the analysis of frequencies displays different results. The probabilities of only one male and five females fall into the interval 1 out of 10 million–1 out of 1 million, whereas all the other individuals belong to the class > 1 out of 1 million.

## Discussion

In the forensic scenario where identification of individuals with identical generic features (e.g., same sex, age, and ancestry) [27, 28] is required, there is the need to overcome the usual parameters of the biological profile and to come up with strategies that would facilitate the identification of the remains [17, 29]. Komar and Lathrop [27] investigated the frequencies of some pathological features in known skeletal collections and suggested caution in using them as personal identifiers. The results drawn from their sample showed that even when multiple fractures, pathological conditions, and surgical procedures are taken into account, they are common and therefore of limited use to ascertain a tentative match (if the features’ presence/absence alone is taken into consideration and compared with known frequencies in a reference group). Similarly, Cappella et al. [29] tested the abovementioned approach on individuals of the same collection as this study, identifying common and less common features that may provide guidance for the search of AM information. Within this research avenue, the use of other morphological features of the skeleton for personal identification can be investigated. As such, this is a first attempt at exploring the potential of cranial non-metric traits for the identification of unknown skeletal remains. Although noted in data collection, non-metric traits are not usually taken into full consideration when elaborating the biological profile; their use for other purposes than biodistance analyses is not well acknowledged and has not been investigated yet. Data collection of non-metric traits (e.g., terminology, scoring) has not been standardized yet; thus, different procedures for scoring, description, and analysis of data were suggested [25]. Furthermore, the uniqueness of these variants cannot be exhaustively assessed because modern population lacks reported frequencies [17]. All these issues lead to inconsistency, methodological discrepancies, and incomparability which make the application of these features to the identification process cumbersome, given that repeatability and reliability of the methods are essential for court admissibility [30, 31].

Subscribing Komar and Lathrop [27], this study does not aim at presenting a new stand-alone method for personal identification, rather suggesting that combinations of non-metric traits might be an additional aspect to consider when comparing AM and PM data. If validated, this approach could be a preliminary step that completes the biological profile of an unknown human cranium and provides the investigators with additional insights. However, the observations would not be based on morphological comparison of skeletal [3, 19] or dental [32, 33] structures, rather on the evaluation of clinical information, calculation of compound probabilities, and comparison with data from known populations, in a similar fashion to identifications based on genetic profiles. Establishing the frequency of skeletal features (e.g., trauma, pathologies, variants) is essential before they can be deemed individualizing and used to calculate probabilities of identity [29, 34]. Watamaniuk and Rogers [35] applied this approach to the morphology of the thoracic vertebral margin. Compound probabilities derived from the frequencies of variants on the vertebral margin were used to assess the strength of potential matches. Following this logic, the present study used frequencies of a set of bilateral non-metric cranial traits and evaluated their potential as personal indicators. Combinations of traits within the class of probability < 1 out of 1 billion could be considered the most useful for personal identification, because it would mean that approximately 7 people all over the world present those exact traits. However, only 10% of the present sample presented such probability. Nonetheless, although most individuals were included in the interval 1 out of 10 million–1 out of 1 million, 25% of the individuals showed that their suite of features can be found with lower probabilities, such as at least 1 out of 100 million. Therefore, the results seem encouraging, as they suggest that some blends of cranial variants can be found with low probabilities in the general population, thus proposing their possible use in the identification procedures. Depending on the AM data available, some cranial traits may be visible or not, according to the type of radiological tests available as antemortem material. This study aimed at verifying the identification potential of a group of 13 variants, potentially visible on a cranium CT scan. However, a recent study pointed out that detection of cranial anatomical variants on CT scan images may be quite difficult, especially those of small size [36]. Again, these shortcomings have some implications for the present study, as the impossibility to reliably recognize some traits on radiographic images significantly may hamper a successful comparison of AM-PM data. However, the traits of this study are among those showing a higher accuracy in Bertoglio et al. [36]. Even though the use of CT scan has recently increased in developed countries [37], this may not be the case of developing countries or people of low economic status all over the world [2, 29], whose limited access to healthcare creates a shortage of AM records and information for the searched individuals, thus hindering the whole identification process.

In order to simulate a possible lack of information deriving from the presence only of conventional X-ray in antero-posterior view, the compound frequencies of a subset of variants that may be visualized on an X-ray were calculated as well. The results show that the potential of cranial traits as personal identifiers is severely limited when a smaller panel of variant is taken into consideration, as almost all the probabilities belong to the class > 1 million. Most compound frequencies appear to be quite high when only seven traits are used for calculation. On one hand, this suggests that a larger panel of variants is more effective to the purpose of identification. On the other hand, the high probabilities from the frequencies of traits visible on an X-ray represent a substantial drawback of this approach, because not all the traits selected may be available on AM material.

However, both for variants assessable from CT scan and conventional X-ray, the potential use for personal identification may be more important in forensic practice than reported by the abovementioned frequencies, for two reasons: first, in some cases the group of possible identity suspect, also called Identification Universe [35], is narrowed down starting from the biological information of the missing, so increasing the applicability also of small frequencies; for instance, in cases of mass disasters involving several hundreds of subjects, also the frequencies that could not be considered sufficient to reach a personal identification according to Earth population may be useful to select a limited number of identity suspect, based on the correspondence of anatomical traits. Secondly, frequencies of anatomical variants may be used not necessarily as a method for conclusive identification, but as a mean to select possible suspects within a large group of possible identities. In the future, the chance of classifying hospital CT scans and filing for each individual the specific set of anatomical variants assessable on radiological analyses may enable to select possible identity suspect, starting from a postmortem set of similar variants. This would have to be further verified through conventional identification methods. Two main issues may influence the frequencies of different sets of variants: possible relationship with age, which means a potential instability of the variant with time, and the trend to bilaterality, which may lead to a duplication of probability. Relationship of variants with other factors, such as age, is not well understood either. Some authors would even suggest not to consider age when studying cranial non-metric traits in adult individuals [38, 39]. This sample revealed that the traits have no relation with age, except for left frontal grooves in males. For this reason, this variant was excluded from the calculation of probability. The analysis of symmetrical expression did not show a relation with laterality for most variants, as well: two exceptions are the mandibular torus and the paracondylar process, which showed a significant relation with bilateral expression, both in males and females. For the mandibular torus, the results are consistent with the literature, which reports a more common bilateral expression [40–42]. The evidence about the effect of side on the paracondylar process is conflicting, as some studies report a prevalence of bilateral processes [43] and others report asymmetry [44]. To the purpose of this study, this evidence could suggest considering the frequency of only one side for the calculation of the compound frequency, in order not to duplicate the frequency and bias the resulting compound data. Nevertheless, a small number of individuals of this sample exhibited a monolateral expression of the trait; hence, it is not recommended to compute only the frequencies of one side, because in these cases considering the frequencies of both sides may improve the identification potential.

Limitations to this study are to be pointed out. First of all, this approach still relies on the availability of AM data, such as CT images showing the relevant traits, which may not be easily accessible. Collection and comparison of PM and AM data may be hampered by several factors. As already mentioned, limited availability of relevant AM information may be related to inaccessible infrastructures, warfare or natural disasters, or because the victims could not access healthcare in their lifetime [2, 27, 29]. Moreover, these observations are based solely on the 100 individuals of the sample. Therefore, in order to comprehensively understand the application of non-metric traits to the identification process, further studies on larger samples should be carried out. In this study, most individuals presented combinations of traits that did not seem rare enough (in the general population) to be deemed individualizing. As shown by considering only some traits visible on an X-ray, a larger panel of variants could be developed and taken into consideration in order to further reduce the frequencies and the probabilities, thus strengthening the value of these traits for identification purposes. In addition, the individuals of this study belong to an Italian population, so the possible influence of ancestry on the distribution of the traits could not be assessed. A suite of features should work better as personal indicators [3, 27, 35]; however, there is still no consensus on how many features should be considered enough to prove a positive identification [35, 45]. This study could not determine a minimum number of traits that would yield a highly probable identification, yet it restates the unspoken rule “the more, the better” [46]. However, this study introduces a new perspective on non-metric traits, which has currently been taken into consideration only for postcranial elements [5, 23, 24]. In some instances, investigations require personal identification to be performed with several approaches, as single-modality identifications may not be successful, and conventional personal identifiers (i.e., genetics, fingerprints, clinical and odontological data) may not be available [47]. If their value for personal identification was validated, non-metric traits may be particularly useful when primary markers of identity cannot be applied. For example, dental treatment has proven an essential tool for personal identification regardless of prevalence [48, 49], although it could come up short when analyzing edentulous individuals.

Future studies on larger samples should also assess the dependence and independence of cranial non-metric traits, in order to calculate compound probabilities of the variants; too little information is currently available about relationships among cranial traits, although Berry and Berry suggested that the traits may derive from independent developmental processes [13]. The authors are aware of the shortcomings related to the limited access to AM records and to the visualization of the traits on CT images that may hamper the application of this approach. In addition, the need for further research on the abovementioned aspects must be fulfilled in order to validate the actual use of these traits for supporting or discarding a suspect of identity. Nonetheless, this study provided new insights into the potential of non-metric traits to personal identification, which is still up for debate.

## Conclusions

This pilot study showed that some combinations of cranial non-metric traits can be found with low probabilities in the general population, thus suggesting their role in providing additional evidence to provisional matches and mismatches. It must be pointed out that this should not be considered a steadfast technique to be used in isolation, rather a supporting tool that may help forensic anthropologists tasked with personal identification of human crania. As this activity hinges on the comparison of AM and PM data, the building block of the identification procedures remains the accessibility to AM records that show the anatomical traits of interest. However, when these data are available, these anatomical variants could be used to narrow down potential matches and strengthen tentative and presumptive identifications.

## Data Availability

Not applicable.
